# Equity and seeking treatment for young children with fever in Nigeria: a cross-sectional study in Cross River and Bauchi States

**DOI:** 10.1186/2049-9957-4-1

**Published:** 2015-01-02

**Authors:** Bikom Patrick Odu, Steven Mitchell, Hajara Isa, Iyam Ugot, Robbinson Yusuf, Anne Cockcroft, Neil Andersson

**Affiliations:** CIET Trust, 71 Oxford Road, Saxonwold, Johannesburg, 2196 South Africa; CIET/PRAM, Department of Family Medicine, McGill University, 5858 Côte-des-Neiges, Suite 300, Montreal, QC H3Z 1Z1 Canada; Community Health Department and Roll Back Malaria Program, Calabar, Cross River State Nigeria; State Ministry of Health, Bauchi, Bauchi State Nigeria; CIET Trust Botswana, PO Box 1240, Gaborone, Botswana

**Keywords:** Severe fever, Malaria, Equity, Access to care, Nigeria

## Abstract

**Background:**

Poor children have a higher risk of contracting malaria and may be less likely to receive effective treatment. Malaria is an important cause of morbidity and mortality in Nigerian children and many cases of childhood fever are due to malaria. This study examined socioeconomic factors related to taking children with fever for treatment in formal health facilities.

**Methods:**

A household survey conducted in Bauchi and Cross River states of Nigeria asked parents where they sought treatment for their children aged 0–47 months with severe fever in the last month and collected information about household socio-economic status. Fieldworkers also recorded whether there was a health facility in the community. We used treatment of severe fever in a health facility to indicate likely effective treatment for malaria. Multivariate analysis in each state examined associations with treatment of childhood fever in a health facility.

**Results:**

43% weighted (%wt) of 10,862 children had severe fever in the last month in Cross River, and 45%wt of 11,053 children in Bauchi. Of these, less than half (31%wt Cross River, 44%wt Bauchi) were taken to a formal health facility for treatment. Children were more likely to be taken to a health facility if there was one in the community (OR 2.31 [95% CI 1.57–3.39] in Cross River, OR 1.33 [95% CI 1.0–1.7] in Bauchi). Children with fever lasting less than five days were less likely to be taken for treatment than those with more prolonged fever, regardless of whether there was such a facility in their community. Educated mothers were more likely to take children with fever to a formal health facility. In communities with a health facility in Cross River, children from less-poor households were more likely to go to the facility (OR 1.30; 95% CI 1.07-1.58).

**Conclusion:**

There is inequity of access to effective malaria treatment for children with fever in the two states, even when there is a formal health facility in the community. Understanding the details of inequity of access in the two states could help the state governments to plan interventions to increase access equitably. Increasing geographic access to health facilities is needed but will not be enough.

**Electronic supplementary material:**

The online version of this article (doi:10.1186/2049-9957-4-1) contains supplementary material, which is available to authorized users.

## Multilingual abstracts

Please see Additional file 1 for translations of the abstract into the six official working languages of the United Nations.

## Background

Malaria remains an important cause of morbidity and mortality worldwide, despite advances in prevention and treatment in recent years [1]. Malaria mortality is concentrated in Africa, accounting for some 90% of all malaria deaths [2]. Children are particularly vulnerable: in endemic countries, malaria may account for 28% of deaths among children under five years of age and 52% of deaths among children aged 5–14 years [3]. A recent systematic review found the risk of malaria was highest among children from the poorest households [4]. The poor are less likely to have access to effective preventive measures [5] and less likely to get effective treatment for malaria [6–8].

While there are many causes of fever in young children, in endemic countries fever in young children is an important indicator of malaria [9], and children with fever should be given prompt treatment with appropriate antimalarial medication, usually artemisinin-based combination therapy (ACT), either after confirmation of the diagnosis or presumptively if diagnostic facilities are unavailable [10]. Studies of parental treatment-seeking behaviour report that most parents in malaria-endemic countries initially treat children with fever at home, often using medications from drug shops [11]. Home treatment is more common in rural areas and among poorer households [7, 12]. Medications from drug shops may often not include effective antimalarials in appropriate doses [11].

Malaria is a major public health problem in Nigeria. The World Health Organization (WHO) World Malaria Report 2012 estimates that Nigeria and the Democratic Republic of the Congo together account for over 40% of the total of malaria deaths globally and, with India, account for 40% of malaria cases worldwide [1]. In Nigeria, malaria may be responsible for 11% of maternal mortality, up to 25% of infant mortality and 30% of under-five mortality [13]. Recent efforts to combat malaria in Nigeria have included distribution of treated bed nets [13, 14], and efforts to ensure prompt treatment of young children with fever with ACT, either after confirmatory tests or presumptively [15].

We hypothesised that in disadvantaged households, children with fever – often an indicator of malaria in endemic areas – would be less likely to be taken for treatment to a facility where they would receive antimalarial medication. As part of a household survey in two states of Nigeria, we examined treatment-seeking behaviour of parents of young children with fever. The objective was to examine the factors, including socioeconomic status and geographic access, associated with taking a child with fever to a formal health facility, where effective antimalarial treatment should be available.

## Methods

**Figure 1:**
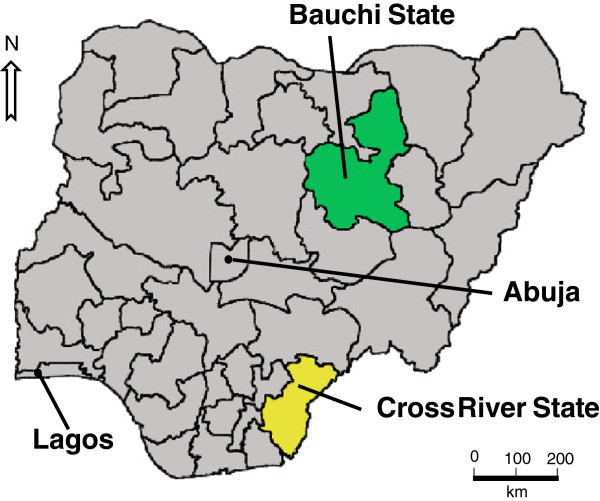
**Map of Nigeria showing the location of Bauchi and Cross River states, as well as the location of Lagos and Abuja.**

Between July and September 2011, a household survey collected information on childhood illnesses and their treatment. This was part of a programme to support evidence-based planning of health services in Cross River and Bauchi states of Nigeria [16, 17]. The two states in the programme were purposively chosen by a national planning group: one from the south (Cross River) and one from the north (Bauchi) (see Figure 1). In the 2006 census, the population in Cross River was 1,471,967 males and 1,421,021 females, while in Bauchi there were 2,369,266 males and 2,283,800 females [18]. Malaria is endemic in both states: throughout the year in Cross River and during the rainy season in Bauchi (May–September) [13].

The stratified last-stage random cluster sample of enumeration areas from the 2006 census comprised 90 enumeration areas (clusters) in each of the two states. The number of urban and rural clusters in the sample in each state reflected the urban/rural balance in the census population. Each cluster comprised contiguous households radiating from a random starting point; interviewers collected data on about 100 children under the age of four years in each cluster.

A one-week training for field teams included both classroom and field practice sessions. Each team included 12 female interviewers, four male members, two supervisors and oversight from a member of the central research team. All the interviewers for the household survey were female; male team members interviewed male household heads outside the homesteads. Each team covered the required number of interviews in each cluster in one or two days per cluster.

The questionnaire for the survey was developed with a design group in each state, which included representatives from the Ministry of Health in each state. A small group of experienced interviewers piloted the instruments several times, and we made adjustments as necessary to improve understanding and flow.

In each household, the female interviewer administered the questionnaire to mothers of children aged 0–47 months. The questionnaire asked mothers about their education, income, and their knowledge about prevention and care of childhood illnesses. It also asked about common illnesses in their young children. In particular, it asked when the child last suffered from severe fever and where the child was taken for treatment of the fever, if anywhere. The field teams also collected information from household heads (or the most senior person present) about household demographics and socioeconomic status. In each community, the team leader established whether there was a formal health facility in the community.

The field team leaders sought consent for the survey from leaders in each community and interviewers sought verbal consent from the head of each household, as well as from each individual respondent. Interviewers did not record any names or identifying information, and did not proceed with any interview unless they could do so without being overheard.

### Analysis

Different operators entered the data twice with validation to minimise keystroke errors using Epi Info™ [19]. The analysis relied on CIETmap open source software [20] that offers a user-friendly interface with the popular statistical programming language R. We calculated weights to correct small differences in urban/rural balance between the sample and the census population in each state; all percentage frequencies presented here are weighted.

We examined associations with the outcome of whether or not the child was taken to a formal health facility for treatment, among children aged 0–47 months who had severe fever in the last month. We restricted analysis to episodes of fever in the last month to increase reliability of recall about a common illness. Severity of fever was according to the perception of the mother. We counted government-run facilities (health posts, primary healthcare centres, comprehensive healthcare centres and hospitals) and formal private facilities as ‘formal health facilities’, but not sources such as pharmacies, chemists and drug sellers. All the formal health facilities should offer effective anti-malarial medication, even though it may not always be available in practice. Potential determinants of the outcome related to the individual child included age and sex and duration of fever (five or more days versus less than five days). Parental characteristics included education of the mother and father. ‘Higher education’ was classified as junior secondary or higher, except for mothers in Bauchi, where few mothers were educated, and for whom ‘higher education’ was *any* formal education. We also noted whether mothers had any incomes of their own. Household characteristics included absolute poverty (very poor households were those reporting they did not have enough food in the last week) and relative poverty (poorer households were those who considered their financial situation to be worse than the average in their community). Other household socioeconomic indicators included: safety of source of drinking water (based on reported source of water); quality of construction (zinc, cement, concrete versus grass, thatch, mud, timber wall construction); number of children (up to two children, or three children or more); sex of the household head; and education of the household head (higher education being junior secondary or higher). We categorised communities according to their location (urban or rural) and whether there was a formal health facility within the community boundaries.

We analysed data from the two states separately. The two states together are not intended to represent the situation in the whole of Nigeria, and the overall project under which the survey was conducted focused on supporting evidence-based health planning at the state level [16, 17]. We examined associations between potential determinants and the outcome in bivariate analysis and then conducted a multivariate analysis using the Mantel-Haenszel procedure [21], adjusted for clustering [22]. Multivariate analysis used backwards deletion from initial saturated models, including all variables significantly associated with the outcome in the bivariate analysis, to produce final models in which all the variables were significantly associated with the outcome. We describe associations using the adjusted odds ratio (ORa) with the cluster adjusted 95% confidence interval (CIca). In the multivariate models for both states, there was interaction between the presence of a formal health facility in the community and other variables, so we created separate models for communities with and without a formal health facility.

We also examined the incremental effects of the socioeconomic factors in the final multivariate models, calculating the proportion of feverish children taken for treatment in a health facility among those with no inequity factors, one inequity factor or two inequity factors, and testing the significance of the trend using the Mantel-extension [23] chi-square for trend.

### Ethical approval

The Ministry of Health in each state gave formal ethical approval for the study (Cross River – CRS/MH/CGS/E-H/018/Vol.1/23, June 23rd 2011; Bauchi MOH/ASS/166/V.1, June 16 2011).

## Results

The field teams collected data on 11,267 children aged 0–47 months in 7,685 households in Cross River, and on 11,277 children aged 0–47 months in 5,535 households in Bauchi. The smaller number of households in Bauchi reflects the larger average size of individual households in this state. Mothers reported that nearly half of the children aged 0–47 months had severe fever in the last month in both Cross River (43% weighted [%wt]; 4,524/10,862) and Bauchi (45%wt; 5,107/11,053). Information about the last episode of severe fever was missing for 405 children in Cross River and 224 children in Bauchi.

Among the children with severe fever in the last month, whose parents reported their treatment, 31%wt (1,536/4,483) in Cross River and 44%wt (2,303/5,038) in Bauchi were taken to a formal health facility for treatment, while about half (49%wt; 2,233/4,483) in Cross River and nearly one third (31%wt; 1,585/5,038) in Bauchi were taken to a pharmacy or a chemist/patent drug seller. These alternative sources of treatment are present in all communities. Some children with severe fever were treated only at home: 15%wt (666/4,483) in Cross River and 22%wt (1,099/5,038) in Bauchi.

Table 1 shows the proportion of children aged 0–47 months with factors potentially related to seeking treatment for severe fever from a formal health facility in both states. Few children were from households reporting food insufficiency in the last week, or considering themselves below average financially, but most were from households with a water source categorised as ‘unsafe’. Education levels of parents and household heads were generally lower in Bauchi than in Cross River. About a quarter of children in Cross River (27%wt) and a third of children in Bauchi (39%wt) lived in communities without a formal health facility in the community.

**Table 1:** **Potential determinants of seeking treatment from a formal health facility, among children aged 0–47 months with severe fever in the last month**

***Potential determinants***	***Weighted % (fraction)***	
	***Cross River state***	***Bauchi state***
Female sex	50 (2,296/4,523)	48 (2,485/5,107)
Fever lasting five or more days	37 (1,674/4,502)	28 (1,351/4,978)
Rural household	72 (3,249/4,524)	83 (4,379/5,107)
Household with three or more children	54 (2,311/4,217)	66 (3,325/5,046)
Household without enough food in the last week	20 (924/4,495)	10 (460/5,074)
Household with unsafe source of drinking water	65 (2,766/4,505)	58 (3,230/5,085)
Female-headed household	18 (811/4,513)	0 (25/5,087)
Household considers financial situation below average	34 (1,593/4,504)	16 (855/5,076)
Household head with less than junior secondary education	40 (1,822/4,436)	75 (3,806/5,054)
Father with less than junior secondary education	30 (1,343/4,303)	76 (3,817/5,048)
Mother with less than junior secondary education	40 (1,832/4,480)	–
Mother with no formal education	–	81 (4,109/5,102)
Mother with no income of her own	42 (1,794/4,252)	36 (1,809/5,045)
Community without a formal health facility	27 (1,116/4,420)	39 (1,875/5,087)

Table 2 shows the results of the bivariate analysis of the factors related to seeking treatment for the feverish child from a formal health facility. Associations significant at the 5% level are in bold. In both states, feverish children were more likely to be taken to a health facility for treatment if they lived in a community with a formal health facility in the settlement. Among feverish children from communities without a formal health facility, 22%wt were taken to a formal health facility for treatment in Cross River and 41%wt in Bauchi. Children from urban communities in Bauchi were more likely to be taken to a formal health facility. Children with fever lasting less than five days were less likely to be taken to a formal health facility. There were associations with several socioeconomic indicators: maternal and paternal education, education of the household head, perceived relative financial situation (Cross River), safety of drinking water source and household food insufficiency.

**Table 2:** **Bivariate associations with seeking treatment from a formal health facility, for children aged 0–47 months with severe fever in the last month**

***Variable***		***Cross River***		***Bauchi***	
		***n/N***	***OR (95% CIca)***	***n/N***	***OR (95% CIca)***
*Sex of child*	Male	767/2,206	1.04	1,191/2,590	1.02
	Female	769/2,276	(0.92–1.18)	1,112/2,448	(0.92–1.14)
*Duration of fever*	≤ 5 days	866/2,800	**0.68**	1,537/3,587	**0.67**
	>5 days	662/1,662	**(0.59–0.78)**	704/1,334	**(0.57–0.79)**
*Number of children in household*	0–2 children	639/1,887	0.96	775/1,701	1.00
	+3 children	796/2,292	(0.83–1.12)	1,490/3,277	(0.89–1.13)
*Sex of household head*	Male	1,280/3,673	1.15	2,286/4,993	1.27
	Female	254/800	(0.93–1.42)	10/25	(0.53–3.01)
*Household food in last week*	Enough	1,260/3,539	**1.39**	2,108/4,551	**1.28**
	Not enough	261/915	**(1.15–1.67)**	183/455	**(1.00–1.64)**
*Safety of water source*	Safe	681/1,720	**1.46**	918/1,830	**1.32**
	Unsafe	850/2,744	**(1.13–1.89)**	1,377/3,186	**(1.04–1.68)**
*Mother has own income*	Yes	891/2,436	**1.29**	1,476/3,192	1.09
	No	550/1,777	**(1.10–1.50)**	788/1,786	(0.92–1.29)
*Perceived relative financial status*	Average or above	1,026/2,884	**1.18**	1,896/4,159	0.96
	Below average	504/1,579	**(1.00–1.38)**	395/848	(0.78–1.19)
*Education of household head*	Junior secondary +	975/2,588	**1.41**	744/1,234	**2.20**
	< junior secondary	541/1,807	**(1.17–1.70)**	1,533/3,751	**(1.78–2.71)**
*Education of the mother*	Junior secondary +	989/2,623	**1.45**	280/423	–
	< junior secondary	535/1,816	**(1.21–1.74)**	2,019/4,610	
*Education of the mother*	Formal education	1,426/4,132	-	606/979	**2.27**
	No formal education	98/307		1,693/4,054	**(1.84–2.78)**
*Education of the father*	Junior secondary +	1,086/2,932	**1.37**	729/1,215	**2.16**
	< junior secondary	401/1,332	**(1.13–1.65)**	1,543/3,764	**(1.75–2.66)**
*Urban/rural community*	Urban	407/1,259	0.89	419/724	**1.77**
	Rural	1,129/3,224	(0.63–1.25)	1,884/4,314	**(1.37–2.29)**
*Type of road to community*	Paved/stoned	1,051/3,085	0.92	859/1,780	1.16
	Dirt	447/1,242	(0.63–1.35)	1,425/3,193	(0.86–1.55)
*Health facility in settlement*	Yes	1,269/3,273	**2.31**	1,533/3,171	**1.33**
	No	239/1,109	**(1.57–3.39)**	763/1,847	**(1.01–1.7)**

Associations significant at the 5% level are shown in **bold**.

Table 3 shows the final multivariate models for factors associated with taking children aged 0–47 months with severe fever in the last month to a formal health facility. The table shows the four final models, for communities with or without a formal health facility in each of the two states. In all four models, children with fever lasting less than five days were less likely to be taken for treatment than those with fever of a longer duration. Mother’s education was positively associated with seeking treatment in each model, except in Cross River communities with a formal health facility. In communities without a formal health facility, mothers who had their own incomes were more likely to seek treatment in a formal health facility in Cross River, but less likely to do so in Bauchi. In Cross River, but not Bauchi, water and food security were associated with seeking treatment in communities with a formal health facility.

**Table 3:** **Final multivariate models of variables associated with taking children, aged 0–47 months, to a formal health facility for treatment of severe fever**

	ORa ^1^	95% CIca for ORa ^2^
**Cross River**		
***Among those from communities with a formal health facility***		
Fever lasted less than five days	0.71	0.61–0.83
Household with enough food in the last week	1.30	1.07–1.58
Household with a safe water source	1.41	1.09–1.82
***Among those from communities without a formal health facility***		
Fever lasted less than five days	0.57	0.41–0.78
Mother has her own income	1.89	1.28–2.78
Mother has junior secondary education or higher	1.54	1.09–2.19
Household head has junior secondary education or higher	2.27	1.55–3.31
**Bauchi**		
***Among those from communities with a formal health facility***		
Fever lasted less than five days	0.63	0.53–0.76
Mother has her own income	1.32	1.06–1.64
Mother has some formal education	1.77	1.37–2.29
Household head has junior secondary education or higher	1.82	1.45–2.28
***Among those from communities without a formal health facility***		
Fever lasted less than five days	0.71	0.53–0.96
Mother has her own income	0.81	0.66–0.99
Mother has some formal education	2.09	1.25–3.50

^1^Adjusted Odds Ratio.

^2^95% Confidence Interval, cluster adjusted.

Table 4 shows the percentages of children with fever taken for treatment with different numbers of the inequity factors from the final multivariate models. In Cross River communities with formal health facilities, a significant compounding of disadvantage was seen between serious poverty (not enough food in the last week) and lack of access to safe water, with the lowest proportion of children with fever taken to a facility for treatment from poor households without access to safe water. In Cross River communities without formal health facilities, and in Bauchi communities with formal health facilities, there was significant compounding of disadvantage between mothers’ lack of education and lack of mothers’ own income; children whose mothers had less education and no income of their own were the least likely to be taken to a health facility for treatment of severe fever.

**Table 4:** **Percentage of children aged 0–47 months taken for treatment in a formal health facility, with increasing numbers of the inequity factors in the final multivariate models in Table** 3

	***% (fraction)***
**Cross River**	
***Among those from communities with a formal health facility*** (chi-square for linear trend 9.41, p <0.05)	
From households with enough food and safe water	46% (442/972)
From households with either enough food *or* safe water	37% (682/1,866)
From households with neither enough food *nor* safe water	32% (132/407)
***Among those from communities without a formal health facility*** (chi-square for linear trend 35.26, p <0.05)	
Mothers have higher education and some income of their own	31% (86/274)
Mothers have either higher education *or* some income of their own	22% (120/537)
Mothers have neither higher education *nor* some income of their own	9% (21/225)
**Bauchi**	
***Among those from communities with a formal health facility*** (chi-square for linear trend 76.55, p <0.05)	
Mothers have some formal education and some income of their own	66% (281/425)
Mothers have either some formal education *or* some income of their own	48% (899/1,877)
Mothers have neither formal education *nor* some income of their own	39% (324/829)
***Among those from communities without a formal health facility*** (chi-square for linear trend 1.72, p =0.19)	
Mothers have some formal education and some income of their own	52% (80/155)
Mothers have either some formal education *or* some income of their own	40% (433/1,092)
Mothers have neither formal education *nor* some income of their own	41% (238/577)

## Discussion

Less than one half of the children under four years of age with severe fever were taken to a formal health care facility in either Cross River State (31%wt) or Bauchi State (44%wt), with even fewer children (22%wt in Cross River and 41%wt in Bauchi) being taken to formal health facilities when these were not readily accessible in the community. Many studies in Nigeria – and other malaria-endemic countries in Africa – have similarly reported that many parents, especially in rural areas, treat young children with fever at home [11, 12, 24, 25]. In our study, parents commonly sought fever medication for children from pharmacies or drug shops: 49%wt in Cross River and 31%wt in Bauchi; this is consistent with findings from other studies [11]. There have been some attempts to train patent drug sellers to treat childhood malaria appropriately and to refer serious cases as necessary [11].

Parents typically use more than one source of treatment for children with fever, starting at home and then seeking care elsewhere if the child’s condition does not improve [26]. In both Cross River and Bauchi, children with fever lasting five days or more were more likely to be taken for treatment to a formal health facility, suggesting that parents take children to these facilities if the fever does not settle. This is a concern since prompt treatment with effective antimalarial medication is important to reduce childhood malaria morbidity and mortality [10].

In Nigeria and elsewhere, geographic access is a well-recognised factor in access to health services, for treatment of malaria as well as other conditions [12, 25, 27, 28]. In both Cross River and Bauchi, we found that children with fever were more likely to be taken to a formal health facility for treatment when there was such a facility in the community. Geographic access was not the only issue; disadvantaged households (those with insufficient food, an unsafe source of water and poorly educated parents or household head) were less likely to take their children with fever to a formal health facility, even in communities with such a facility nearby.

Lack of education of the mother or the household head reduced the chances of a feverish child being taken to a formal health facility. Other authors have reported that children of mothers with less education or knowledge about malaria are less likely to receive appropriate treatment for malaria [29]. A body of literature links malaria to poverty. Poor children are less likely to have access to malaria prevention measures and are more likely to suffer from malaria [4–6]. The poorest households are less likely to take children with fever to a health facility where they can have access to effective antimalarial medication [7, 8, 27]. This reflects a generally lower use of formal health services among the poorest households [30]. In our study, we used the lack of a safe water supply and food insufficiency in the preceding week as indicators of household poverty. Household food insufficiency can be a useful indicator of serious poverty in African and other developing countries, and has been reported to be associated with a number of adverse outcomes [31, 32]. We found that household poverty was a significant factor limiting the use of formal health facilities for a feverish child in circumstances where geographic access was not an issue: in Cross River communities with a health facility.

Poverty, lack of education and other vulnerabilities are not mutually exclusive; compounding inequities may lead to even lower access to services [33]. In the present study, the inequity factors affecting a feverish child’s chances of being taken to a health facility for treatment compounded one another. In both Cross River and Bauchi, we found a decreasing proportion of children being taken for treatment of fever as inequities compounded. This sort of analysis is not often reported, but it is helpful when communicating about findings with planners and policymakers. Policymakers need to understand that households with multiple risk factors are particularly vulnerable and plan accordingly.

An important advantage of this study is that it collected information from a sizeable representative sample in two individual states. This was helpful when discussing the findings to support planning at state level. Members of the study teams of personnel seconded from the state governments discussed findings with stakeholders in the health sector in each state, including policymakers, health workers, civil society groups, non-governmental organisations, traditional leaders, religious leaders and community members. Dissemination tools included district and state scorecards of the results, and docudramas with local actors to share the evidence and stimulate discussion about possible solutions, including addressing inequities in use of formal health services.

### Limitations

This was a cross-sectional study. We can describe associations but cannot draw strong conclusions about causality. In this study, the outcome (seeking care in a formal health facility) cannot have preceded potential determinants (such as education level and socioeconomic status of parents). But we may not have accounted for all potential confounders in the described associations. Severe fever was as perceived by the mothers (who may have under or over-estimated severity), but this is unlikely to have affected associations with treatment seeking for severe fever. As with all questionnaire surveys, there are a number of potential biases, arising both from the respondents and from the interviewers. We reduced these as far as possible by careful design and piloting of the questionnaire, and by careful training and supervision of the interviewers.

## Conclusion

Our findings emphasise the inequity of access to effective malaria treatment for children with fever in two states of Nigeria. Understanding the details of inequity of access in the two states can help the respective state governments to plan interventions to increase access equitably. Increasing geographic access is needed but will not be enough.

## Electronic supplementary material

Additional file 1: **Multilingual abstracts in the six official working languages of the United Nations.** (PDF 326 KB)
