# Supplementary material for: Equity and seeking treatment for young children with fever in Nigeria: a cross-sectional study in Cross River and Bauchi States
Source: Infect Dis Poverty. 2015 Jan 2;4(1):1. doi: 10.1186/2049-9957-4-1 (PMC4322819; doi:10.1186/2049-9957-4-1)
Supplement: Supplementary file 1 — Additional file 1: Multilingual abstracts in the six official working languages of the United Nations. (PDF 326 KB) [file 40249_2014_88_MOESM1_ESM.pdf]

Translation of the abstract into the six official working languages of the United Nations

العدالة والمطالبة بعلاج للأطفال الصغار المصابين بالحمى في نيجيريا: دراسة عرضية في ولايتي كروس ريفر وباوتشي

بيكوم باتريك أودو، ستيفن ميتشيل، هجارا عيسى، إيام أوجت، روبنسون يوسف، آن كوكروفت، نيل آندرسون

الملخص

الخلفية: الأطفال الفقراء لديهم خطر أعلى للإصابة بالملاريا ومن المحتمل تلقيهم القليل من العلاج الفعال. وتعتبر الملاريا أحد أهم أسباب الإصابة بالأمراض والوفاة عند الأطفال النيجيريين والعديد من حالات حمى الطفولة نتيجة الإصابة بالملاريا. وتناولت هذه الدراسة العوامل الاجتماعية والاقتصادية المرتبطة باصطحاب الأطفال المصابين بالحمى لتلقي العلاج بالمرافق الصحية.

طرق العمل: سأل مسح أسري أجري في ولايتي باوتشي وكروس ريفر النيجيريتين الآباء أين سعوا لعلاج أطفالهم الذين تتراوح أعمارهم بين 0-47 شهر ويعانون من حمى شديدة خلال الشهر الماضي وتم جمع معلومات عن الوضع الاجتماعي والاقتصادي للأسر. سجل الباحثون كذلك ما إذا كان هناك مرفق صحي حيث يقيمون. واستخدمنا علاج الحمى الشديدة في المرفق الصحي لنوضح بشكل كبير العلاج الفعال للملاريا. وتناول التحليل متعدد المتغيرات في كل ولاية مدى الارتباط بعلاج حمى الطفولة في المرفق الصحي.

النتائج: من 10,862 طفل شملهم المسح في كروس ريفر، هناك 43٪ أصيبوا بحمى شديدة خلال الشهر الماضي، و 45٪ أصيبوا بها من 11,053 طفل شملهم المسح في باوتشي. من هؤلاء، أقل من النصف (34٪ كروس ريفر، 46٪ باوتشي) تم أخذهم إلى مرفق صحي رسمي للعلاج. وكان الأطفال أكثر عرضة ليؤخذوا إلى مرفق صحي إذا تواجد بمحل إقامتهم (النتيجة العامة 2.31 [95٪ مدى الثقة 1.57-3.39] في كروس ريفر، النتيجة العامة 1.33 [95٪ مدى الثقة 1.0-1.7] في باوتشي). والأطفال المصابون بحمى تدوم أقل من خمسة أيام كانوا أقل عرضة ليؤخذوا للعلاج من أولئك الذين لديهم حمى لفترة أطول، بغض النظر عما إذا كان هناك مثل هذا المرفق بمحل إقامتهم. كانت الأمهات المتعلمات أكثر ميلاً لأخذ الأطفال المصابين بالحمى إلى المرفق الصحي الرسمي. في المجتمعات التي لديها مرفق صحي في كروس ريفر، كان أطفال الأسر الأقل فقراً أكثر ميلاً للذهاب إلى هذا المرفق (النتيجة العامة 1.30؛ 95٪ مدى الثقة 1.07-1.58).

الاستنتاج: هناك عدم عدالة في الوصول إلى العلاج الفعال للملاريا للأطفال المصابين بالحمى في الولايتين، حتى في وجود مرفق صحي رسمي بمحل إقامتهم. وفهم تفاصيل عدم العدالة في الولايتين يمكن أن يساعد حكومة الولايتين لتخطيط التدخلات من أجل زيادة فرص الوصول بشكل عادل. ويعتبر زيادة وسائل الوصول الجغرافي للمرافق الصحية مطلوباً ولكنه سيظل غير كاف.

Translated from English version into Arabic by Saher Salama, through

TRANSLATORS
WITHOUT BORDERS

尼日利亚克里斯河州（Cross River）和包奇州（Bauchi states）关于发热儿童就医及其公平性的横断面调查

Bikom Patrick Odu, Steven Mitchell, Hajara Isa, Iyam Ugot, Robbinson Yusuf, Anne Cockcroft, Neil Andersson

摘要

引言：贫困儿童感染疟疾的风险更高，并且更可能无法获得有效的治疗。疟疾是尼日利亚儿童发病和死亡的重要病因之一，许多儿童发热是由疟疾引起的。本研究旨在调查影响发热儿童到正规卫生机构就诊的相关社会经济因素。

方法：在尼日利亚克里斯河州（Cross River）和包奇州（Bauchi states）开展入户调查，询问家长在过去一个月中 0～47 月龄发热患儿在何处就诊，并收集家庭的社会经济状况信息，同时记录社区中卫生机构的情况，并把严重发热在卫生机构就诊作为有效治疗疟疾的指标。采用多因素分析研究各州家庭社会经济状况与发热儿童在卫生机构就诊情况的关系。

结果：克里斯河州调查了 10,862 位儿童，43%患儿在过去一个月中严重发热。包奇州 11,053 位患儿中有 45%严重发热。在正规卫生机构就诊的患儿不到一半。其中，社区中有 1 家卫生机构的社区儿童更可能到卫生机构就诊（克里斯河州：OR 2.31 [95%CI 1.57–3.39]；包奇州：OR 1.33 [95%CI 1.0–1.7]）；持续发热少于 5 天的儿童与发热时间更长的相比不太可能去就诊，且与社区中是否有卫生机构不相关。受过教育的母亲更可能带孩子去正规卫生机构就诊。而在克里斯河州那些有 1 个卫生机构的社区中，来自较贫困家庭的儿童更可能到卫生机构就诊（OR 1.30； 95%CI 1.07–1.58）。
结论：该两个州发热儿童得到疟疾有效治疗的机会不平等，甚至在那些有正规卫生机构的社区中也是如此。了解这些不平等性的细节将有助于政府规划公平性提高的干预措施。越来越多的地方仅需要卫生机构是不够。

Translated from English version into Chinese by Yin Jian-hai, through

CHINESE CENTER FOR DISEASE CONTROL AND PREVENTION
NATIONAL INSTITUTE OF PARASITIC DISEASES

Equité et recherche de traitement pour les jeunes enfants ayant de la fièvre au Nigeria: une étude transversale dans les états de Cross River et de Bauchi.

Bikom Patrick Odu, Steven Mitchell, Hajara Isa, Iyam Ugot, Robbinson Yusuf, Anne Cockcroft, Neil Andersson

Résumé

Contexte: Les enfants défavorisés courent un plus grand risque de contracter la malaria et ont moins de chances de recevoir un traitement efficace. La malaria est une cause importante de morbidité et de mortalité chez les enfants nigériens, et de nombreux cas de fièvre infantile sont dus à la malaria. Cette étude a examiné les facteurs socio-économiques en rapport avec le traitement des enfants fiévreux dans des établissements de santé traditionnels.
Méthodes: Une enquête sur les ménages, menée dans les états nigériens de Cross River et de Bauchi, a permis de demander aux parents où ils recherchaient un traitement pour leurs enfants de 0 à 47 mois présentant une forte fièvre le mois dernier ; cette enquête a recueilli les informations sur le statut socio-économique des ménages. Les travailleurs sociaux ont également recherché s'il y avait un établissement de santé dans la communauté. Nous avons utilisé un traitement contre les fortes fièvres dans un établissement de santé pour indiquer un traitement pouvant être efficace contre la malaria. Dans chaque état, des analyses multivariées ont étudié des associations avec les traitements contre la fièvre infantile préconisés dans un établissement de santé.
Résultats: Sur les the 10.862 enfants examinés à Cross River, 43% avaient une forte fièvre au cours du mois dernier, et sur les 11.053 enfants examinés à Bauchi, 45% présentaient cet état fiévreux. Parmi ceux-ci, moins de la moitié (34% pour Cross River, 46% pour Bauchi) a été transportée dans un établissement de santé traditionnel, lorsqu'il y en avait un dans la communauté (OR 2.31 [95%CI 1.57–3.39] à Cross River, OR 1.33 [95%CI 1.0–1.7] à Bauchi). Les enfants dont la fièvre a duré moins de cinq jours ont été moins sujets à recevoir le traitement que ceux dont la fièvre se prolongeait, qu'il y ait eu ou non un tel établissement dans leur communauté. Les mères instruites ont eu davantage tendance à emmener leurs enfants fiévreux dans un établissement de santé traditionnel. Dans les communautés possédant un établissement de santé à Cross River, les enfants des ménages moins défavorisés étaient davantage sujets à être conduits dans cet établissement (OR 1.30; 95%CI 1.07–1.58).
Conclusion: Il y a une inégalité d'accès à un traitement efficace contre la malaria pour les enfants fiévreux dans deux états, même lorsqu'il existe un établissement de santé traditionnel dans la communauté. Une bonne compréhension des détails de l'inégalité d'accès dans les deux états pourraient aider les gouvernements de l'état à planifier des

interventions pour accroître équitablement l'accès. Accroître l'accès géographique aux établissements de santé est nécessaire, mais ne sera pas suffisant.

Translated from English version into French by Ode Laforge, through

Равенство и обращение за лечением маленьких детей, страдающих жаром, в Нигерии: перекрестное исследование в штатах Кросс-Ривер и Баучи

Биком Патрик Оду, Стивен Митчел, Хаджара Иса, Иям Угот, Робинсон Юсуф, Анн Коккрофт, Нейл Андерсон

Краткий обзор

История вопроса: Дети из бедных слоев населения подвержены более высокому риску заражения малярией и имеют меньше возможностей получения эффективного лечения. Малярия является одной из главных причин заболеваемости и смертности среди нигерийских детей, и в большинстве случаев причиной жара является малярия. Данное исследование рассматривает социоэкономические факторы, влияющие на лечение детей с жаром в медицинских учреждениях.

Методы: В рамках опроса семей, который был проведен в нигерийских штатах Баучи и Кросс-Ривер, родителям задали вопрос о том, обращались ли они за медицинской помощью для своих детей в возрасте от 0 до 47 месяцев, в случае если дети испытывали жар в течение последнего месяца. Также собиралась информация о социоэкономическом положении семей. Кроме того, была собрана информация о наличии в населенных пунктах медицинских учреждений. Лечение очень высокой температуры, которое проводилось в медицинском учреждении, с большой долей вероятности рассматривалось как эффективное лечение малярии. Многомерный анализ рассмотрел факторы, влияющие на вероятность лечения жара у детей в медицинском учреждении.

Результаты: Из 10862 детей, принявших участие в опросе в штате Ривер-Кросс, у 43 % был жар в течение последнего месяца, а в штате Баучи у 45 % из 11053 детей. Из них менее чем половина (34 % в Кросс-Ривер и 46% в Баучи) прошла лечение в медицинском учреждении. При наличии медицинского учреждения в населенном пункте вероятность обращения в такое учреждение была выше (отношение шансов 2,31 [95% доверительный интервал 1,57–3,39] в Кросс-Ривер, отношение шансов 1,33 [95% доверительный интервал 1,0–1,7] в Баучи). Родители детей, у которых жар продолжался в течение более чем 5 дней, реже обращались за медицинской помощью, чем в случаях, когда жар продолжался более длительное время, независимо от наличия медицинского учреждения в населенном пункте. Матери, имеющие образование, чаще обращались в медицинские учреждения в случае жара у детей. В населенных пунктах Кросс-Ривер, в которых были медицинские учреждения, дети из более обеспеченных семей чаще получали помощь в медицинском учреждении (отношение шансов 1.30; 95% доверительный интервал 1,07–1,58).

Заключение: Существует неравный доступ к эффективному лечению малярии у детей в двух рассмотренных штатах даже в тех случаях, когда в населенном пункте есть медицинское учреждение. Понимание факторов, влияющих на неравный доступ в этих двух штатах, может помочь правительствам штатов при планировании

мероприятий по повышению равенства доступа к медицинской помощи. Расширение охвата медицинскими учреждениями в географическом отношении является недостаточным.

Translated from English version into Russian by Natalia.A, through

Equidad y búsqueda de tratamiento para niños con fiebre en Nigeria: estudio transversal en los estados de Bauchi y Cross River

Bikom Patrick Odu, Steven Mitchell, Hajara Isa, Iyam Ugot, Robbinson Yusuf, Anne Cockcroft, Neil Andersson

Resumen

Antecedentes: Los niños pobres corren mayor riesgo de contraer la malaria y tienen menor probabilidad de recibir un tratamiento eficaz. La malaria es una causa importante de morbilidad y mortalidad entre los niños nigerianos y muchos casos de fiebre infantil se deben a ella. El presente estudio analiza los factores socioeconómicos relacionados con llevar a los niños con fiebre a centros de salud oficiales para su tratamiento.

Métodos: En una encuesta realizada entre familias de los estados de Bauchi y Cross River en Nigeria, se preguntó a los padres dónde buscaron tratamiento para sus hijos de edades comprendidas entre los 0 y los 47 meses cuando sufrían de fiebre grave y se recogió información acerca del estatus socioeconómico dichas familias. Los investigadores en el terreno también registraron si existía un centro de salud en la comunidad. Utilizamos el tratamiento para la fiebre grave en un centro de salud con el fin de señalar un probable tratamiento eficaz para la malaria. El análisis multivariante analizó la relación del tratamiento con la fiebre infantil en un centro de salud en cada uno de los estados.

Resultados: De los 10.862 niños encuestados en Cross River, el 43% sufrió de fiebre grave durante el último mes, así como el 45% de los 11.053 encuestados en Bauchi. De ellos, se llevó a un centro de salud oficial para su tratamiento a menos de la mitad (el 34% en Cross River y el 46% en Bauchi). La probabilidad de llevar al niño al centro de salud aumentaba si existía uno en la comunidad (OR 2,31[95%CI 1.57–3.39] en Cross River, OR 1,33 [CI 1,0-1,7] en Bauchi). La probabilidad de llevar a los niños con fiebre con una duración menor de cinco días era más remota que aquellos con fiebre más prolongada, sin importar si existía o no tal centro en su comunidad. Las madres con estudios eran más propensas a llevar al niño con fiebre a un centro de salud oficial. Los niños procedentes de hogares con mayores recursos económicos en comunidades con centro de salud en Cross River tenían más probabilidades de acudir al centro (OR 1.30; 95%CI 1.07–1.58).

Conclusión: Existe desigualdad de acceso a un tratamiento eficaz contra la malaria para niños con fiebre en los dos estados, aún cuando hay un centro de salud oficial en la comunidad. Comprender los detalles de la desigualdad de acceso en ambos estados ayudaría a los gobiernos de los mismos a planificar intervenciones para aumentar el acceso equitativamente. El aumento del acceso geográfico a los centros de salud es necesario pero no suficiente.

Translated from English version into Spanish by Raquel Bentué, through
